# Assessment of Computed Tomography Perfusion Research Landscape: A Topic Modeling Study

**DOI:** 10.3390/tomography9060158

**Published:** 2023-11-01

**Authors:** Burak B. Ozkara, Mert Karabacak, Konstantinos Margetis, Vivek S. Yedavalli, Max Wintermark, Sotirios Bisdas

**Affiliations:** 1 Department of Neuroradiology, MD Anderson Cancer Center, 1400 Pressler Street, Houston, TX 77030, USA; 2 Department of Neurosurgery, Mount Sinai Health System, 1468 Madison Avenue, New York, NY 10029, USA; 3 Russell H. Morgan Department of Radiology and Radiological Sciences, Johns Hopkins Hospital, 600 N Wolfe Street, Baltimore, MD 21287, USA; 4 Department of Neuroradiology, The National Hospital for Neurology and Neurosurgery, University College London NHS Foundation Trust, London WC1N 3BG, UK; 5 Department of Brain Repair and Rehabilitation, Queen Square Institute of Neurology, University College London, London WC1N 3BG, UK

**Keywords:** CT perfusion, topic modeling, natural language processing

## Abstract

The number of scholarly articles continues to rise. The continuous increase in scientific output poses a challenge for researchers, who must devote considerable time to collecting and analyzing these results. The topic modeling approach emerges as a novel response to this need. Considering the swift advancements in computed tomography perfusion (CTP), we deem it essential to launch an initiative focused on topic modeling. We conducted a comprehensive search of the Scopus database from 1 January 2000 to 16 August 2023, to identify relevant articles about CTP. Using the BERTopic model, we derived a group of topics along with their respective representative articles. For the 2020s, linear regression models were used to identify and interpret trending topics. From the most to the least prevalent, the topics that were identified include “Tumor Vascularity”, “Stroke Assessment”, “Myocardial Perfusion”, “Intracerebral Hemorrhage”, “Imaging Optimization”, “Reperfusion Therapy”, “Postprocessing”, “Carotid Artery Disease”, “Seizures”, “Hemorrhagic Transformation”, “Artificial Intelligence”, and “Moyamoya Disease”. The model provided insights into the trends of the current decade, highlighting “Postprocessing” and “Artificial Intelligence” as the most trending topics.

## 1. Introduction

The quantity of scholarly articles is consistently increasing, demonstrating a yearly growth rate of 4% in publications and 1.8% in the number of references per publication [1]. The continuous increase in scientific output makes it challenging for researchers, as they need significant time to gather and understand these results. Given the imperative for medical practitioners and researchers to remain abreast of contemporary research and literature in order to deliver the most current care and information to patients and advance scientific knowledge, there exists a demand for enhanced methods of research synthesis that optimize efficiency. In addressing this need, the topic modeling approach emerges as a novel solution. It provides a way to discern hidden themes within a large research landscape, paving the path for recognizing research trends and granting valuable insights [2]. These tools are crucial in identifying both rising and waning areas in medical research and making the exhaustive process of reviews more manageable.

Computed tomography perfusion (CTP) imaging has become a prominent diagnostic tool. CTP has established itself as the favored advanced imaging modality in many stroke clinical trials, solidifying its standing [3]. The integration of CTP into the standard operational procedures of stroke centers worldwide is becoming more prevalent, serving as a testament to its effectiveness and reliability. The increasing popularity of CTP can also be attributed to its notable diagnostic accuracy in detecting myocardial ischemia. It has been observed that CTP exhibits a diagnostic accuracy that is comparable to stress magnetic resonance imaging and positron emission tomography perfusion [4]. Moreover, the capability of CTP to evaluate vascularization in tumor tissues without invasive procedures enhances its scope of utility, thus demonstrating its versatility and significance in contemporary medicine [5]. Given the rapid advancement and widespread implementation of CTP, we have recognized the importance of undertaking a topic modeling endeavor. The objective of our study was to gain an understanding of the dynamic research landscape pertaining to CTP.

## 2. Materials and Methods

We conducted a search of the Scopus database from 1 January 2000 up to 16 August 2023, using the keywords “computed tomography perfusion”, “CT perfusion”, “perfusion CT”, and “perfusion computed tomography” in article titles and keywords to identify relevant articles. The article types considered for inclusion in this study were limited to “Article” and “Review”. Only articles written in English were included. These specific article types, along with their corresponding metadata elements, such as article title, abstract, year, and citation count, were subsequently obtained. We removed articles lacking abstracts to ensure that our topic modeling accurately captured the essential themes, as abstracts provide a concise summary of an article’s main content and findings. The articles were classified into distinct quartiles (Q1, Q2, Q3, and Q4) based on the number of citations they received, with the division determined by the 25th percentile for each quartile.

In our study, we employed BERTopic, a topic modeling technique that facilitates the interpretation of topics by preserving important words in topic descriptions [6]. The Bidirectional Encoder Representations from Transformers (BERT) embeddings were generated utilizing the S-PubMedBert-MS-MARCO model, which has been specifically optimized for the medical domain [7]. Upon acquiring the embeddings, we eliminated stop words that lacked contextual significance in the text, such as “the” and “of”. The utilization of the Natural Language Toolkit library in the Python programming language enabled the attainment of this objective [8]. In the model, the parameters for both the minimum topic size and the number of extracted words per topic were configured to be 50. The former establishes the minimum permissible magnitude for a subject, whereas the latter specifies the quantity of words extracted from each subject. Using the BERTopic model, we derived a collection of topics along with their respective representative articles. To assign labels to these topics, both authors (B.B.O. and M.K.) reached a mutual agreement based on an analysis of keywords and representative articles. We also crafted word clouds to visualize the primary keywords associated with these topics. Subsequently, we investigated the distribution of these leading topics across various citation quartiles and publication years.

For the current decade, the 2020s, we set out to analyze prevailing trends. Linear regression models were the primary tools to discern these trends within topics [9]. By focusing on linear patterns, we enhanced the methodology, simplifying the interpretation of our results. During this analysis, topic probabilities, publication years, and topic names were drawn from the dataset. Here, the topic probability represented the chance of an article aligning with a specific topic due to its content. Afterward, we computed the average topic probability for each year and topic. With these consolidated data, linear regression models were built for every unique topic, facilitating the distinction between trending (hot) and waning (cold) topics. Linear regression models were constructed using mean topic probability as the dependent variable and the publication year as the independent variable. A “hot topic” denotes an area of research showing a positive trend in topic probability over time, indicating growing interest and relevance within the research community. In contrast, a “cold topic” refers to subjects displaying a negative trend in topic probability, signifying diminishing interest or relevance in that particular research area. The model’s source code can be found in the project’s GitHub repository (https://github.com/mertkarabacak/TopicModeling_CTP).

## 3. Results

The initial dataset consisted of 3562 articles; by limiting our scope to solely the article types of “Article” and “Review”, a total of 995 articles were excluded. The lack of abstracts resulted in the additional exclusion of 53 articles. Out of the total of 2514 articles that were analyzed, a classification process was conducted on 2356 articles, resulting in their categorization into 12 distinct categories. Consequently, the 158 articles comprising the remaining subset were identified as outliers due to their inability to be assigned to any specific category.

Table 1 displays all the crafted topics, each characterized by its distinct set of keywords and indicating the total count of articles associated with each topic. The topics that were crafted encompass “Tumor Vascularity”, “Stroke Assessment”, “Myocardial Perfusion”, “Intracerebral Hemorrhage”, “Imaging Optimization”, “Reperfusion Therapy”, “Postprocessing”, “Carotid Artery Disease”, “Seizures”, “Hemorrhagic Transformation”, “Artificial Intelligence”, and “Moyamoya Disease”.

**Figure 1:**
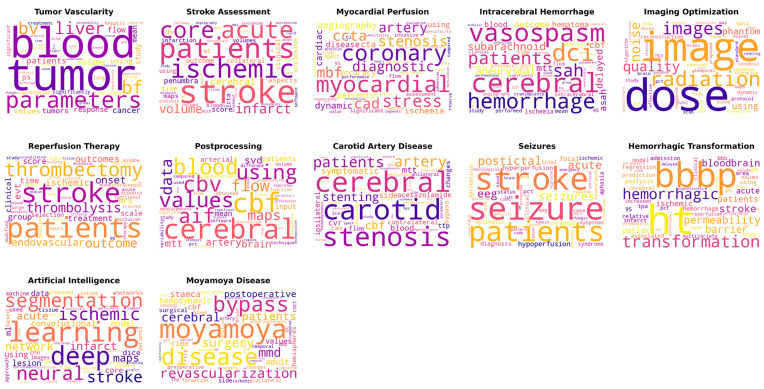
Word clouds representing each topic, where the size of each keyword indicates its frequency.

Figure 1 presents word clouds corresponding to individual topics, which consist of keywords that symbolize the fundamental concepts and components. Within these word clouds, the prominence of each keyword reflects its frequency, offering a concise overview of the primary themes related to each topic.

**Figure 2:**
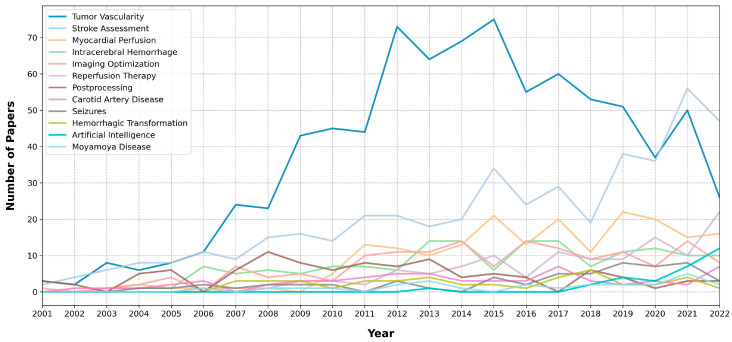
Prominence of the topics based on the publication year.

We illustrated the progression of these topics by charting the count of papers based on their publication year (Figure 2). This chart captures the annual changes in topic prominence.

**Figure 3:**
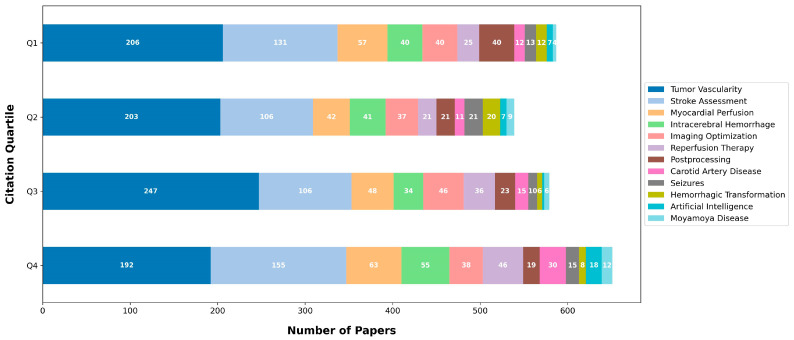
Citation quartiles.

The analysis presented in Figure 3 illustrates the quartiles of citations for the topics.

**Figure 4:**
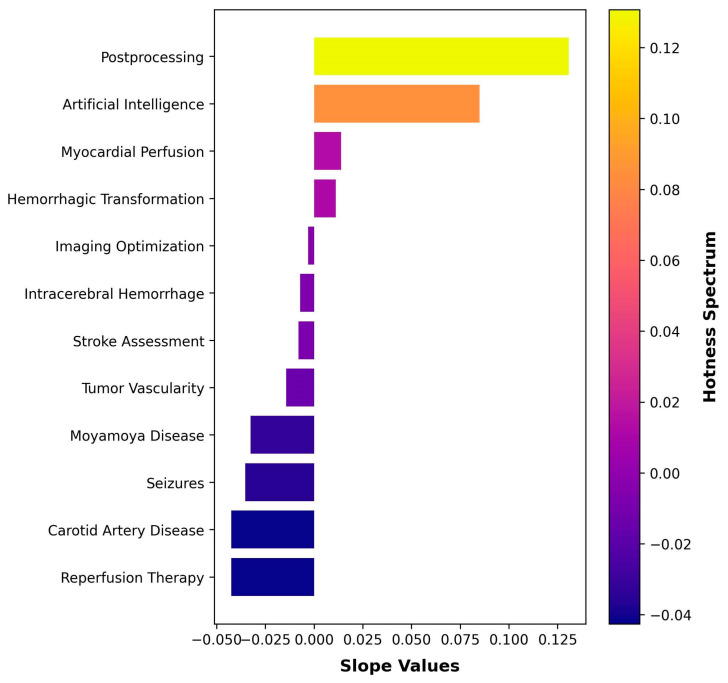
Trends in this decade.

The model offered insights into the current decade’s patterns, spotlighting “Postprocessing” and “Artificial Intelligence” as the most prominent subjects. “Myocardial Perfusion” and “Hemorrhagic Transformation” were among the hot topics as well. Conversely, the topics identified as least prevalent in this decade were “Carotid Artery Disease” and “Reperfusion Therapy”. We visualized these patterns using a color-gradient bar chart in Figure 4.

## 4. Discussion

The extensive and rapidly expanding body of scholarly literature highlights the essential significance of topic modeling in effectively navigating and integrating research findings. In our research, we employed BERTopic to analyze 2514 articles pertinent to CTP, successfully identifying 12 central topics: “Tumor Vascularity”, “Stroke Assessment”, “Myocardial Perfusion”, “Intracerebral Hemorrhage”, “Imaging Optimization”, “Reperfusion Therapy”, “Postprocessing”, “Carotid Artery Disease”, “Seizures”, “Hemorrhagic Transformation”, “Artificial Intelligence”, and “Moyamoya Disease”. Leveraging linear regression models allowed us to discern the dominant research trajectories manifesting in the current decade. The examination of the development of these topics revealed that “Postprocessing” and “Artificial Intelligence” have ascended as the foremost subjects in the 2020s, with “Myocardial Perfusion” and “Hemorrhagic Transformation” also gaining prominence. Conversely, the interest in “Carotid Artery Disease” and “Reperfusion Therapy” seems diminishing, as evidenced by their declining trends. Our method offers a distinct viewpoint and essential insights, becoming a significant resource for researchers specializing in CT Perfusion, offering a valuable glimpse into the dynamic progression of the CTP research domain.

The topic “Tumor Vascularity” emerged as the most dominant, in part because stroke-related subjects were divided into smaller subcategories. CTP helps to evaluate the vitality and extent of tumor vascularization. CTP has revealed marked disparities in perfusion values between normal and neoplastic tissues, with notably elevated perfusion metrics observed in patients with head and neck, rectal, hepatic, and lung masses [10]. Consequently, this assessment plays a pivotal role in diagnosing and staging, prognosticating, and tracking responses to therapies [11]. In more specific terms, for certain tumor types, CTP has proven effective in differentiating between benign and malignant processes [12,13,14], forecasting potential metastatic events [15], predicting therapeutic responses [16,17], and monitoring responses to various treatments [18,19,20,21,22]. CTP also expands its utility in distinct areas, such as assessing the effects of radiation therapy on the hemodynamics of the spinal cord [23]. Given these applications of CTP, it is understandable that “Tumor Vascularity” stands out as a significant topic in CTP research literature. Although CT imaging has been clinically proven to be capable of discerning tumor perfusion, vessel morphology, and response to therapy, its popularity in this field has decreased, according to our analysis (Figure 2). This is likely due to concerns about the additional radiation exposure associated with perfusion acquisition techniques and limitations in the use of iodinated contrast agents. As a result, magnetic resonance imaging (MRI) and ultrasound-based perfusion assessments have become more favored, especially in cases where high-resolution soft-tissue anatomical imaging is needed (such as MRI for the brain) or point-of-care assessment is necessary (such as ultrasound for the liver). However, the implementation of CTP in whole-body staging CT protocols can be seamless. Additionally, there is less variation in scanning parameters and postprocessing methodologies compared to MRI, and the output of quantitative imaging biomarkers is a strong advantage for the further use and development of CTP, particularly in conjunction with low-dose protocols.

In our study, “Stroke Assessment” emerged as the second most prevalent topic. This is notably significant, considering that the model subdivided stroke-related topics into various subcategories during the analysis. CTP plays a key role in evaluating patients presenting with acute stroke symptoms. Ischemic stroke occurs when a cerebral artery becomes blocked, the primary source of disability in the US [24]. This blockage triggers the unavoidable demise of a section of the brain tissue called the core infarction. Concurrently, there exists another segment of the brain that, although deprived of sufficient blood supply, remains alive; this area is widely known as the penumbra [25]. The penumbra is vulnerable to permanent damage if the resumption of blood circulation is not promptly facilitated. Consequently, the main objective of reperfusion therapy during the management of ischemic strokes is to safeguard the penumbra by re-establishing the flow of blood through the arteries [25]. The procedure of CTP is utilized to conduct a thorough assessment of the brain’s parenchymal tissue amidst instances of cerebral ischemia. This method can be employed to ascertain the extent of core infarction as well as the surrounding penumbra area. Despite its unambiguous utility in detecting oligemic and penumbra tissue, there are concerns about the associated contrast and radiation exposure accompanying a CTP study. Hence, in the initial hours post-stroke, CTP is primarily reserved for instances where noncontrast CT and CTA fail to provide adequate diagnostic clarity [26]. However, the American Heart Association guidelines advocate using CTP or MR imaging in triaging patients beyond the 6 h mark [27]. This change in stance arises since the efficacy of endovascular therapy between 6 and 24 h has been demonstrated in patients selected through CTP [28,29]. Furthermore, the findings from these two pivotal trials published in 2018 could plausibly account for the rising prevalence of the “Stroke Assessment” topic since 2018. Another application of CTP in stroke assessment involves evaluating the cerebral perfusion status and reserve in patients with Moyamoya disease [30,31]. This chronic condition is characterized by occlusion and stenosis of the cerebrovascular system, potentially leading to cerebral ischemia and hemorrhage [32]. Given these multifaceted considerations, it is understandable that “Stroke Assessment” emerged as a dominant topic in our study. It is worth noting that the keyword “stroke” also appears in the “Seizures” topic. Differentiating clinically between acute ischemic stroke and epileptic seizure can be challenging. Making the correct diagnosis can prevent unnecessary reperfusion therapy, and CT perfusion may play a pivotal role in this distinction, which explains the presence of the “stroke” keyword in the topic.

“Myocardial Perfusion” emerged as the third most prevalent topic in our study. The process of CTP imaging involves assessing myocardial perfusion under both rest and hyperemic circumstances [33]. CTP is conducted following the introduction of iodinated contrast, where the left ventricular myocardium is captured during the first pass of the contrast bolus passing through [33]. Capturing images during the beginning stage of the first passage of the contrast is vital because of the rapid washout [34]. In studies conducted at single centers, myocardial CTP imaging has exhibited impressive accuracy when benchmarked against modalities like single-photon emission CT, cardiovascular magnetic resonance, invasive coronary angiography, positron emission tomography, and invasive fractional flow reserve [35]. While the application of CTP for myocardial perfusion has been somewhat constrained, primarily because coronary CT angiography on its own offers a substantial negative predictive value in ruling out myocardial ischemia, its relevance emerges in scenarios demanding clarity on ischemia presence [35]. This is particularly true in cases with coronary artery stenoses of ambiguous hemodynamic impact, pronounced coronary calcification, or coronary stents [35,36,37]. In our study, “Myocardial Perfusion” secured the third position, a reflection of its varied uses encompassing static, dynamic, and dual-energy acquisitions. Additionally, “Myocardial Perfusion” maintained its position as one of the hot topics of this decade, emphasizing the growing interest in utilizing CTP in this domain.

Another interesting topic created by our topic modeling analysis, emerging as the 5th most prevalent topic, was “Imaging Optimization”. This concept mainly encompasses strategies for minimizing radiation exposure without compromising the precision of perfusion parameters [25,38]. Furthermore, it involves approaches that fine-tune the initial analysis of CTP first-pass analysis, optimize the timing of the contrast administration, and hone image acquisition techniques [39,40]. Additionally, it considers the finer details, such as adjusting image-acquisition parameters and optimizing z-direction coverage and section thickness to procure the highest quality images [40]. Noise reduction efforts have also been a significant area of research for this topic [41,42,43]. This concerted effort to optimize imaging procedures represents a significant stride in advancing the field, merging safety with efficiency and accuracy.

The heightened emphasis on “Postprocessing” and “Artificial Intelligence” in our trend analysis for the 2020s indicates a shift in CTP research directions. Several factors may potentially explain why “Postprocessing” has emerged as a hot topic in recent research. Firstly, the variability in postprocessing represents a significant challenge in myocardial CTP implementation, potentially spurring heightened investigative interest [35]. Additionally, in acute ischemic stroke, the decision for endovascular intervention is frequently based on CTP analysis. This involves quantifying penumbra and infarct core using perfusion parameter thresholds, which unfortunately lack consistency across various vendors, further driving research in this area [44]. Numerous studies have also delved into comparing diverse postprocessing software [45,46,47]. Thus, the surge in focus on “Postprocessing” may be attributed to the ongoing quest for a unified postprocessing method. Meanwhile, artificial-intelligence-driven solutions are infiltrating every industry, radiology being no exception [48]. In line with this, artificial intelligence has emerged as a prominent theme in CTP research, showcasing numerous applications in both stroke and myocardial perfusion, aligning well with our topic modeling findings [49,50,51,52,53].

“Hemorrhagic Transformation” was also one of the hot topics of this decade, highlighting the increasing use of CTP in addressing this dangerous complication in patients with acute ischemic stroke [54]. The hemorrhagic infarction that arises following venous or arterial thrombosis and embolism is known as hemorrhagic transformation [55]. Hemorrhagic transformation exhibits a wide array of severity levels, from minor pinpoint bleeding within the affected tissue to a substantial hematoma that sprawls past the infarcted area’s edges [56]. This transformation’s radiological categorization stemmed from the initiatives taken by the European Cooperative Acute Stroke Study (ECASS), which discerns between various forms: minor petechial hemorrhagic infarction (HI1), extensive petechial hemorrhagic infarction (HI2), modest parenchymal hemorrhage (PH1) (constituting less than 30% of the infarct with a slight mass effect), and significant parenchymal hemorrhage (PH2), which accounts for more than 30% of the infarct and exhibits a pronounced mass effect [57]. In the past, various imaging techniques such as noncontrast CT and MRI have been explored to anticipate the occurrence of hemorrhagic transformation. However, throughout the past ten years, numerous researchers have examined the potential of CTP in forecasting hemorrhagic transformation during acute ischemic strokes, which is consistent with the trends identified in our analysis [58]. CTP serves as a valuable tool in predicting hemorrhagic transformation, a potentially life-threatening complication associated with reperfusion therapies [55]. Various perfusion parameters are known to correlate with hemorrhagic transformation, including time to peak, relative cerebral blood flow, relative mean transit time, relative cerebral blood volume, and time to maximum [59,60,61,62]. In their detailed meta-analysis on using CTP to predict hemorrhagic transformation in acute ischemic stroke, Suh et al. studied 15 articles, which included data from a total of 1134 patients. They found a pooled sensitivity and specificity of 84% and 74%, respectively. Additionally, the area under the hierarchical summary receiver operating characteristic curve was 0.84 [58]. Furthermore, the state of hypoperfusion is recognized to have a connection with hemorrhagic transformation. This was evidenced by Suh et al.’s findings which indicated lower CBV and CBF levels, along with extended Tmax values, in individuals who underwent hemorrhagic transformation compared to those who did not [58]. Therefore, the existing data support the utilization of CT perfusion in forecasting hemorrhagic transformation in cases of acute ischemic stroke.

Our study is not without limitations. First and foremost, while we employed a solid NLP approach, the effectiveness of our topic modeling hinges on the accuracy and comprehensiveness of the metadata extracted from different publications. Another potential limitation of our study is the possibility of encountering duplicate articles or those that have been updated over time in the Scopus database, as referenced in prior research [63]. Moreover, our assessment of trends was confined to linear trajectories, potentially overlooking the intricate nuances of the shifting research landscape. Additionally, we intentionally excluded keywords like “CTP” or “PCT” to avoid incorporating numerous irrelevant articles into the model, which could skew the outcomes. Our approach aimed to strike a balance between including a wide range of articles and excluding irrelevant ones.

## 5. Conclusions

In conclusion, our study offers valuable insights into the evolving landscape of CTP research. Using natural language processing and topic modeling, we were able to identify and track the major topics discussed in recent literature. The dominant topics were tumor vascularity, stroke assessment, and myocardial perfusion. In the meantime, postprocessing and artificial intelligence emerged as prominent trends, highlighting a shift toward optimizing analysis techniques. The trends and themes identified by our computational analysis align well with advances and innovations in the field of CTP. As the technique continues to gain traction for a variety of diagnostic applications, additional research will be required to address current limitations, standardize acquisition and postprocessing protocols, and maximize the potential of artificial intelligence. Our study provides a high-level overview of the activity in this field, laying the groundwork for more in-depth investigations on specific topics and applications.

## Figures and Tables

**Table 1:** Topics, keywords, number of articles, and representative articles.

Topic Label	Key Words	Number of Articles	Representative Articles
Tumor Vascularity	tumor, blood, parameters, bf, liver, bv, patients, cancer, volume, flow, values, using, tumors, study, mean, ps, response, significantly, hepatic, significant, correlation, lung, carcinoma, treatment, group, permeability, compared, hcc, analysis, time, arterial, respectively, pancreatic, higher, value, ml100, performed, tissue, pct, lesions, changes, mtt, cell, showed, different, 005, dynamic, used, underwent, groups	848	Title: Perfusion computed tomography for monitoring induction chemotherapy in patients with squamous cell carcinoma of the upper aerodigestive tract: Correlation between changes in tumor perfusion and tumor volume
Stroke Assessment	stroke, patients, ischemic, acute, core, infarct, volume, cerebral, penumbra, aspects, score, outcome, time, collateral, clinical, occlusion, maps, cta, infarction, using, volumes, ml, cbv, blood, analysis, ncct, software, angiography, followup, circulation, tissue, mismatch, thresholds, ais, early, dwi, noncontrast, rapid, 95, onset, used, study, cbf, final, included, treatment, within, vessel, good, correlation	498	Title: Quantifying infarct core volume in ischemic stroke: What is the optimal threshold and parameters of computed tomography perfusion?
Myocardial Perfusion	myocardial, coronary, stress, stenosis, cad, ccta, mbf, diagnostic, artery, angiography, dynamic, cardiac, cta, ischemia, disease, patients, accuracy, using, significant, 95, assessment, spect, invasive, ffr, ci, flow, performance, rest, reserve, value, compared, study, specificity, analysis, adenosine, combined, sensitivity, fractional, respectively, heart, evaluation, detection, segments, myocardium, quantitative, obstructive, hemodynamically, cardiovascular, reference, alone	210	Title: Dynamic myocardial CT perfusion imaging-state of the art
Intracerebral Hemorrhage	cerebral, vasospasm, dci, hemorrhage, patients, sah, subarachnoid, aneurysmal, delayed, cbf, asah, mtt, outcome, blood, hematoma, early, ischemia, mean, time, intracerebral, flow, study, 95, pressure, performed, angiography, cranioplasty, clinical, ich, perihematomal, brain, ci, group, aneurysm, spot, value, infarction, parameters, days, analysis, values, cbv, sensitivity, significantly, volume, within, expansion, dsa, deficits, transit	170	Title: Relationship between vasospasm, cerebral perfusion, and delayed cerebral ischemia after aneurysmal subarachnoid hemorrhage
Imaging Optimization	dose, image, radiation, images, quality, noise, phantom, using, reconstruction, lowdose, data, dynamic, protocol, maps, reduction, cerebral, contrast, scan, algorithm, brain, temporal, tube, values, doses, mgy, study, proposed, compared, time, scans, pct, mas, iterative, effective, cbf, flow, blood, used, standard, sampling, model, purpose, skin, quantitative, injection, mean, acquisition, clinical, stroke, exposure	161	Title: Temporal feature prior-aided separated reconstruction method for low-dose dynamic myocardial perfusion computed tomography
Reperfusion Therapy	patients, stroke, thrombectomy, thrombolysis, outcome, endovascular, onset, ischemic, evt, outcomes, treatment, time, group, score, mrs, scale, clinical, selection, intravenous, hours, window, acute, large, treated, modified, functional, core, rankin, occlusion, nihss, study, mismatch, 90, therapy, baseline, safety, reperfusion, wakeup, mechanical, days, volume, criteria, median, recanalization, selected, mortality, beyond, tissue, groups, vs	128	Title: Utilization of CT perfusion patient selection for mechanical thrombectomy irrespective of time: A comparison of functional outcomes and complications
Postprocessing	cerebral, cbf, blood, cbv, values, using, aif, flow, data, maps, artery, svd, mtt, brain, patients, mean, input, arterial, quantitative, function, obtained, volume, technique, pct, study, analysis, time, compared, different, measurements, variability, used, software, algorithm, postprocessing, algorithms, stenosis, deconvolution, parameters, correlation, differences, transit, stroke, significant, mca, regions, singular, images, mrp, decomposition	103	Title: Differences in CT perfusion maps generated by different commercial software: Quantitative analysis by using identical source data of acute stroke patients
Carotid Artery Disease	cerebral, carotid, stenosis, patients, artery, stenting, cbf, symptomatic, mtt, cvr, blood, side, acetazolamide, ipsilateral, bypass, contralateral, cas, ttp, flow, changes, unilateral, challenge, ica, occlusion, hemodynamic, cerebrovascular, internal, spect, study, hps, parameters, severe, asymptomatic, time, middle, impairment, test, group, disease, mean, surgery, bto, mca, chronic, hyperperfusion, cbv, significant, 0001, brain, ischemic	68	Title: Carotid artery stenting and blood–brain barrier permeability in subjects with chronic carotid artery stenosis
Seizures	seizure, stroke, patients, postictal, acute, eeg, hypoperfusion, focal, hyperperfusion, pct, diagnosis, aphasia, ictal, epileptic, syndrome, cerebral, epilepticus, case, ischemic, symptoms, status, clinical, may, neurological, emergency, cortical, mimics, left, blood, brain, aura, se, patterns, isolated, handl, cases, pres, deficits, pattern, encephalopathy, migraine, code, changes, onset, epilepsy, presented, study, reversible, strokelike	59	Title: Acute Ischemic Stroke or Epileptic Seizure? Yield of CT Perfusion in a “Code Stroke” Situation
Hemorrhagic Transformation	ht, bbbp, transformation, hemorrhagic, permeability, stroke, barrier, bloodbrain, patients, acute, ischemic, patlak, hemorrhage, analysis, ph, regression, model, using, ps, area, prediction, relative, 95, associated, admission, tpa, infarct, bbb, cerebral, multivariate, values, pct, clinical, increased, study, reperfusion, thrombolysis, volume, delayed, risk, ais, parenchymal, higher, nlr, parameters, significantly, therapy, intracerebral, blood, hc	46	Title: Hemorrhagic transformation of ischemic stroke: Prediction with CT perfusion
Artificial Intelligence	learning, deep, segmentation, neural, ischemic, stroke, network, infarct, maps, acute, convolutional, lesion, using, model, ml, core, data, dice, networks, cnn, machine, based, tissue, used, images, proposed, volume, patients, approach, 4d, penumbra, time, spatiotemporal, image, prediction, unet, trained, coefficient, performance, predict, absolute, algorithm, training, mean, software, parameter, segment, achieve, compared, challenge	34	Title: Prediction of Stroke Infarct Growth Rates by Baseline Perfusion Imaging
Moyamoya Disease	moyamoya, disease, bypass, revascularization, surgery, mmd, cerebral, patients, postoperative, stamca, hemodynamic, adult, cbf, values, time, surgical, hemispheres, side, preoperative, dt, rttp, mms, brain, rcbf, collateral, rmtt, formation, changes, temporal, blood, ttp, volume, operation, months, atherosclerotic, seconds, artery, ischemic, mtt, relative, improved, compared, hemodynamics, significant, flow, wbctp, underwent, 005, significantly, combined	31	Title: CT perfusion assessment of Moyamoya syndrome before and after direct revascularization (superficial temporal artery to middle cerebral artery bypass)

## Data Availability

No new data were created or analyzed in this study. Data sharing is not applicable to this article.
